# Supplementary material for: Bovine Coronavirus Prevalence and Risk Factors in Calves on Dairy Farms in Europe
Source: Animals (Basel). 2024 Sep 23;14(18):2744. doi: 10.3390/ani14182744 (PMC11429389; doi:10.3390/ani14182744)
Supplement: Supplementary file 1 [file animals-14-02744-s001.zip › animals-3208940-supplementary.pdf]

Table S1

Univariable farm clustered logistic regression analysis of variables of herd management and health factors associated with BCoV shedding in neonatal and weaned calves.

NC = neonatal calves, WC = weaned calves, Code = Variable code, Description = short description of variable. -- = variable not tested, * = P-value ≤ 0.10, ** = P-value ≤ 0.05.

NC	WC	Code	Description
**	n.s.	Q15	Lactating Herd Size
--	--	Q16	Number of dry cows
--	**	Q17	Number of weaned non-pregnant heifers
--	--	Q18	Number of pregnant heifers
**	n.s.	Q19	Number of preweaned calves
--	--	Q20	Number of insemination bulls
**	*	Q21	Total number of cattle on farm
n.s.	n.s.	Q22	Overall yearly replacement percentage
n.s.	n.s.	Q23	% of heifers replacement internal
**	n.s.	Q24	% of heifers purchased
--	--	Q25	Breed: Indicate breeds
**	**	Q27b	MYPerCow_305d +/- median
n.s.	n.s.	Q29	Milk Fat %
n.s.	n.s.	Q30	Milk Protein %
n.e.	**	Q31	Herd Average bulk milk SCC
n.s.	**	Q31b	BTM_SCC +/- median
n.s.	n.s.	Q32	% cows with mastitis per lactation
**	n.s.	Q33	Average age (in months) of heifer insemination
**	n.s.	Q34	Average age (in months) at first calving
**	n.s.	Q36	% calves with diarrhoea first week
**	n.s.	Q37	% calves with diarrhoea in preweaning period
**	n.s.	Q38w	Week when diarrhoea starts (w1, w2 or w3+)
n.s.	*	Q40	Prophylaxis against cryptosporidiosis
	n.s.	Q42	% BRD in preweaning calves
n.s.	**	Q44	Do you vaccinate the calves against BRD?
--	n.s.	Q46	% of calves with BRD post-weaning to 6 months
**	**	Q47	Vaccination of dams for calf enteric disease?
**	n.s.	Q49	Vaccination of dams for calf BRD?
n.s.	n.s.	Q51	Diagnostics for diarrhoea
**	**	Q53	Enteric disease outbreaks in adult cattle within the last year
n.s.	**	Q54	Enteric disease outbreaks in adult cattle within the last 5 years
**	**	Q55	BRD outbreak within the last year
**	**	Q56	BRD outbreak within the last 5 years
n.s.	n.s.	Q57	Liters of colostrum fed in the first 24 hours?
**	n.s.	Q58b	Transition milk feeding (yes/no)

**	--	Q59fresh	Whole milk feeding to calves
**	--	Q59waste	Waste milk: Code -1 = MR only, 0= fresh milk, 1= high SCC milk, 2 = post ABtx milk, 3 = ABtx milk
**	n.s.	Q61	Pasteurized milk or waste milk
**	n.s.	Q62	Calf milk replacers (CMR)
n.s.	n.s.	Q63	Age (days) when CMR are introduced
**	n.s.	Q64	Average daily feed (L) in the 1st month?
**	n.s.	Q64b	1st Month: 0: ≤ = 6 L, 1: > 6 L
--	n.s.	Q65	Average daily feed (L) in the 2nd month?
**	n.s.	Q65b	2nd Month: 0: ≤ = 6 L, 1: > 6 L
--	n.s.	Q66	Average age (in days) at weaning
--	**	Q66b	Weaning age +/- 70 days
**	*	Q67	Daily milk/CMR feeding frequency
	*	Q68	Average age (in days) when calves are placed in group pens
**	**	Q68w	NC; Week 1, week 2, week3 or more, WC before versus after 8 weeks
*	n.s.	Q69	# of calves in first group pen
**	n.s.	Q70	Automatic milk feeding equipment
n.s.		Q71	Average stocking density in preweaning group pens
	n.s.	Q72	Average stocking density in group pens within 1 month postweaning
**	**	Cow_fn	Average % positive samples in fresh cows

Table S.2

Univariable farm clustered logistic regression analysis of variables of biosecurity (Biocheck) factors associated with BCoV shedding in neonatal and weaned calves.

NC = neonatal calves, WC = weaned calves, Code = Variable code, Description = short description of variable. -- = variable not tested, * = P-value ≤ 0.10, ** = P-value ≤ 0.05.

NC	WC	Code	Biocheck Question Description
n.s.	n.s.	Biocheck_ext	External score
n.s.	**	Biocheck_int	Internal score
n.s.	n.s.	Biocheck	Total score
**n.s.**	**n.s.**	**BC_A**	**A. Purchase and reproduction**
n.s.	n.s.	BC1_Cattle_purchased	1. Are cattle being purchased?
n.s.	*	BC1_Purchase_freq	sum of purchase of cows, youngstock and bulls
n.s.	n.s.	BC8	8. Are all new cattle put into quarantine?
n.s.	n.s.	BC15	15. Are there any cattle that leave the farm and return afterwards?
**	**	BC17_Repro	AI, AI&bull, bull
******	**n.s.**	**BC_B**	**B. Transport and carcass removal**
n.s.	n.s.	BC20	20. Do all vehicles have to pass through clean transport baths before entering the farm?
**	n.s.	BC21	21. Do external transport vehicles and transporters have access to any of the areas where cattle are kept?
**	n.s.	BC23	23. Is the transport vehicle for the cattle empty on arrival at the farm?
n.s.	n.s.	BC24	24. Is the transport vehicle always cleaned and disinfected before entering the farm?
n.s.	n.s.	BC25	25. Is there a separate carcass storage space with a hard surface floor present?
n.s.	n.s.	BC27	27. Is the carcass storage space protected from vermin, cats and dogs?
n.s.	*	BC28	28. Can the carcasses be removed by the rendering company without them entering the premises of the farm?
n.s.	n.s.	BC29	29. Are carcasses manipulated with gloves, or are hands cleaned and disinfected after manipulation of carcasses?
n.s.	n.s.	BC30	30. Is all the material used for the manipulation of carcasses cleaned and disinfected?
**n.s.**	**n.s.**	**BC_C**	**C. Feed and water**
n.s.	**	BC31	31. Are the feed storage facilities (e.g. ensilaged feed, feed mixer, concentrates, …) protected from pets and vermin?
n.s.	n.s.	BC32	32. Are feeding utensils used only for feed (e.g there's no double use for manure)?

n.s.	n.s.	BC33	33. Is the quality of the drinking water checked every year at the source or at the storage tank by means of a bacteriological analysis?
n.s.	**	BC34	34. Is the quality of the drinking water checked every year at the main outlets (i.e. where the cattle drink) by means of a bacteriological analysis?
**n.s.**	**n.s.**	**BC_D**	**D. Visitors and farmworkers**
n.s.	n.s.	BC35	35. Are visitors obliged to notify you of their presence before entering the stables (e.g. visitor's register)?
n.s.	n.s.	BC36	36. Is there a separate space available for changing boots and clothes and washing hands/putting on gloves?
n.s.	n.s.	BC37	37. Are there any farmworkers who also work at (or frequently visit) other farms?
n.s.	n.s.	BC40	40. Does the artificial insemination technician come to the farm?
n.s.	n.s.	BC42	42. Does the cattle salesman come to the farm?
n.s.	n.s.	BC44	44. Does the hoof trimmer come to the farm?
n.s.	**	BC46	46. Are there any other visitors (e.g. feed supplier, advisors, milk collector, any others) that enter the farm and come into contact with the cattle?
**n.s.**	**n.s.**	**BC_E**	**E. Vermin control and other animals**
n.s.	*	BC48	48. Is an insect control programme present on the farm?
n.s.	n.s.	BC49	49. Is a rodent control programme present on the farm?
n.s.	n.s.	BC50	50. Is a bird control programme present on the farm (e.g. netting to keep birds out)?
n.s.	n.s.	BC51	51. Do your cattle, including the youngstock, have access to the outside?
n.s.	n.s.	BC54	54. Are there other commercially exploited cattle present on the farm?
n.s.	n.s.	BC56b	56. Are any other farm animals being kept? (No versus Yes)
n.s.	**	BC57	57. Do pets have access to the stables? Only cats versus none
n.s.	n.s.	BC58	58. Is manure from other farms being spread on farmlands within a 500-metres radius (0.3 miles) of your farm and pastures?
n.s.	**n.s.**	**BC_F**	**F. Health management**
n.s.	n.s.	BC59	59. Are the sick cattle physically isolated from the healthy cattle?

n.s.	*	BC66	66. Are the sick cattle taken care of before or after the healthy cattle?
n.s.	n.s.	BC67	67. Can a unit of sick cattle be completely separated from the other cattle in case of a disease outbreak?
n.s.	n.s.	BC68	68. Is a register with the animal health data being kept?
n.s.	n.s.	BC69	69. Are there written protocols for vaccination, disease treatment and hygiene procedures?
n.s.	n.s.	BC71	71. Are there dedicated injection needles that are specific to each age group available?
n.s.	**n.s.**	**BC_G**	**G. Calving management**
n.s.	n.s.	BC72b	72. Are there maternity pens and/or a box for C-sections available on the farm? Individual versus group
n.s.	*	BC73	73. Is the maternity pen either ever used to house the sick cattle or is the maternity pen adjacent to the sick cattle?
n.s.	n.s.	BC74	74. Are cattle in the maternity pen fully separated from the other animals?
n.s.	**	BC77b	77. When helping with the calvings/abortions, are the hands and the used obstetric materials always cleaned and disinfected before and after each calving/abortion?
n.s.	*	BC78	78. Are the cow's hindquarters (including the udder) always cleaned and disinfected before each calving?
n.s.	n.s.	BC79	79. When does the separation of the calf from the mother take place?
n.s.	n.s.	BC80	80. If an abortion takes places, is the cow tested afterwards (i.e. abortion protocol)?
n.s.	n.s.	BC81	81. Where are the foetal membranes and tissues disposed of after a calving/abortion?
**n.s.**	**n.s.**	**BC_H**	**H. Calf management**
n.s.	n.s.	BC82	82. How many <u>litres</u> of colostrum are administered to the calf within the first six hours of birth?
n.s.	n.s.	BC83	83. Pasteurized versus raw
n.s.	**	BC84	84. Is it checked if the colostrum quality is sufficient?
n.s.	*	BC85	85. Is there a frozen or artificial reserve of colostrum present, in case that either the mother does not provide enough milk or the colostrum is of insufficient quality?

n.s.	**	BC87b	87. Are the materials used for colostrum administration (e.g. tubes, bottles, etc.) cleaned and disinfected after each use?
n.s.	n.s.	BC88	88. Are the calves housed in individual calf boxes/hutches or separate areas?
*	n.s.	BC89	89. Are the individual calf boxes/hutches/separate areas empty after each use?
*	n.s.	BC90b	90. Are the individual calf boxes/hutches/separate areas ... before each new introduction of calves?
n.s.	n.s.	BC91	91. Is contact possible with calves in different hutches/boxes/separate areas?
n.s.	n.s.	BC92	92. Are milk feeding buckets/teats reused between calves during the same feeding session?
n.s.	n.s.	BC93	93. Are the calves ever fed with waste milk (i.e. milk that is not suitable for the milk tank)?
n.s.	n.s.	BC94	94. Are the feeding buckets cleaned after each feeding?
n.s.	**	BC_I	I. Dairy management
n.s.	**	BC_J	J. Adult cattle management
n.s.	n.s.	BC115A	115.1. How often <u>a year</u> is the adult stable cleaned?
n.s.	n.s.	BC115B	115.2. How often <u>a year</u> is the adult stable disinfected?
n.s.	n.s.	BC117	117. Do the cows have to regularly pass through a hoof disinfection footbath?
n.s.	*	BC_K	K. Working organisation and equipment
n.s.	n.s.	BC118	118. Are the cattle grouped per age category in the stable?
n.s.	*	BC119	119. Has a full separation between age groups been established?
n.s.	**	BC121	121. Is farm work performed in a specific order?
n.s.	*	BC123	123. Is there any material being shared with other farms that enters the stables and/or has contact with your cattle?

Table S3. Dairy herds enrolled in this study, including lactating herd size, neonatal calves (NC#), weaned calves (WC#) and fresh cows (FC#) sampled, bulk tank milk antibody (BTM ab) (%inh) and mean serum antibody (% inh) for all cattle sampled (Cattle ab), neonatal calves (NC ab), weaned calves (WC ab) and fresh cows (FC ab), the percentage of animals per category shedding BCoV (NC BCoV+, WC BCoV+ & Fc BCoV+) and the Biocheck scores (total%, external% and internal%).

Country	Farm	Lact herd size	BTM ab	Cattle ab	NC ab	WC ab	FC ab	NC #	NC BCoV+	WC #	WC BCoV+	FC #	FC BCoV+	BioCheck Total %	BioCheck external %	BioCheck internal %
AT	AT1	58	91,9	79,6	85,1	60,9	92,8	6	0%	12	9%	7	0%	56	74	37
AT	AT2	80	88,4	57,5	51,9	60,5	60,2	4	50%	12	0%	60	0%	57	83	31
AT	AT3	38	88,0	59,2	68,1	43,3	66,1	6	67%	6	33%	10	0%	63	81	45
AT	AT4	68	90,4	29,9	33,8	16,1	39,8	6	0%	5	0%	10	0%	63	77	49
AT	AT5	71	89,0	51,3	61,7	35,1	57,0	7	7%	9	11%	14	0%	53	53	53
AT	AT6	80	89,2	73,3	89,7	39,3	90,8	9	0%	6	0%	12	0%	54	66	41
AT	AT7	120	90,3	68,8	66,5	69,9	70,1	10	25%	11	14%	11	0%	62	77	46
AT	AT8	51	89,6	60,0	49,2	62,7	68,0	8	50%	3	0%	11	0%	52	68	36
AT	AT9	44	72,9	30,6	45,3	4,9	41,7	10	0%	6	0%	10	0%	60	80	40
AT	AT10	322	61,0	74,4	87,1	52,8	83,4	10	0%	10	15%	10	0%	63	86	40
BE	BE1	220	89,6	51	64	14	75	9	0%	10	5%	6	0%	55	73	37
BE	BE2	337	93,4	61	58	47	79	19	37%	15	70%	14	11%	49	62	40
BE	BE3	155	87,7	59	83	18	77	14	0%	13	0%	9	0%	50	71	29
BE	BE4	227	87,9	45	51	28	56	21	48%	15	10%	11	14%	59	77	41
BE	BE5	136	87,4	66	57	66	74	10	0%	9	0%	8	6%	70	85	54
BE	BE6	80	84,6	62	55	64	67	5	100%	5	90%	5	20%	59	70	47

Country	Farm	Lact herd size	BTM ab	Cattle ab	NC ab	WC ab	FC ab	NC #	NC BCoV+	WC #	WC BCoV+	FC #	FC BCoV+	BioCheck total	BioCheck external	BioCheck internal
BE	BE7	320	94,6		76	51		5		11				53	66	40
BE	BE8	210	92,7	59	50	60	66	10	65%	10	25%	5	0%	59	72	45
BE	BE9	180	96,1	73	83	56	78	11	18%	12	50%	5	10%	57	72	41
BE	BE10	218	94	70	75	52	81	10	0%	10	0%	8	0%	57	70	43
CZ	CZ1	574	75,2	48,5	35,0	47,8	62,7	20	0%	20	0%	10	0%	64	77	50
CZ	CZ2	589	87,3	47,5	46,6	43,5	52,4	20	0%	20	13%	10	0%	65	75	54
CZ	CZ3	263	89,8	50,9	47,8	26,0	78,8	20	3%	20	65%	10	0%	70	88	52
CZ	CZ4	644	92,8	74,8	73,5	66,0	84,9	20	0%	20	5%	10	0%	71	77	65
CZ	CZ5	323	93,6	69,2	66,9	57,9	82,8	20	28%	20	55%	10	0%	61	65	56
CZ	CZ6	443	92,4	54,9	49,0	46,8	69,0	20	23%	20	25%	10	5%	61	72	50
CZ	CZ7	932	92,5	60,5	53,9	51,2	76,4	20	0%	20	30%	9	0%	58	70	46
DK	DK1	640	86,6	42	37,8	36,3	52,1	12	83%	10	5%	8	0%	47	64	30
DK	DK2	144	53,5	32	26,3	17,1	52,8	10	20%	12	0%	6	0%	58	76	40
DK	DK3	167	82,6	41	55,6	10,6	57,8	11	0%	10	0%	10	0%	55	73	36
DK	DK4	450	76,6	67	73,3	42,6	83,8	15	0%	12	58%	10	0%	58	76	40
DK	DK5	1400	81,7	44	33,7	45,4	54,3	16	50%	12	33%	6	17%	58	76	40
DK	DK6	175	83,9	60	62,4	66,9	49,4	13	46%	12	25%	10	0%	50	74	26
DK	DK7	246	92,7	57	52,8	35,7	83,2	12	0%	12	21%	7	0%	49	64	34
ES	ES2	1813	83,2	69,7	82,1	42,0	85,1	15	0%	15	0%	7	0%	72	82	62
ES	ES3	190	67,2	81,1	60,0	96,9	86,5	6	0%	10	0%	5	0%	52	70	34
ES	ES4	280	75,9	47,6	45,0	55,5	42,2	11	41%	10	10%	7	0%	46	54	38
ES	ES5	320	25,2	66,4	74,8	61,1	63,4	10	40%	10	65%	11	0%	53	59	46
ES	ES6	470	75,3	74,1	78,2	55,5	88,6	10	0%	10	5%	5	0%	71	85	56

Country	Farm	Lact herd size	BTM ab	Cattle ab	NC ab	WC ab	FC ab	NC #	NC BCoV+	WC #	WC BCoV+	FC #	FC BCoV+	BioCheck total	BioCheck external	BioCheck internal
ES	ES7	115	73,0	50,8	38,9	48,8	64,8	7	0%	16	3%	11	0%	59	74	44
FR	FR2	147	94,7	75,7	75,1	71,1	80,9	10	70%	10	15%	9	11%	55	68	41
FR	FR4	100	86,5	59,3	56,2	58,3	63,3	10	60%	10	0%	10	0%	54	68	39
FR	FR5	89	92,6	53,0	38,3	65,0	55,7	10	10%	9	11%	9	0%	62	67	56
FR	FR6	77	91,0	16,4	3,8	2,0	43,6	8	0%	10	0%	10	5%	59	72	46
FR	FR7	100	90,5	39,6	25,7	41,0	52,2	10	95%	10	55%	10	20%	36	36	35
FR	FR8	129	88,0	27,7	34,16	3,57	45,41	10	0%	9	0%	10	0%	62	72	51
FR	FR9	115	84,0	37,2	36,7	27,1	47,9	10	0%	10	5%	10	0%	47	61	33
FR	FR10	100	92,5	42,7	37,1	55,4	35,7	10	30%	10	10%	10	10%	51	67	34
GR	GR1	480	92,9	68,1	59,6	52,9	91,7	12	58%	12	17%	5	10%	67	81	52
GR	GR2	320	90,0	30,7	17,5	24,0	50,7	10	0%	10	0%	5	0%	63	76	49
GR	GR3	134	90,0	51,8	60,0	49,1	46,4	10	0%	10	0%	5	0%	55	78	32
GR	GR4	180	94,8	50,4	55,06	42,9	53,2	10	10%	10	65%	5	20%	56	77	34
GR	GR5	282	90,6	30,7	15,85	25,1	51,22	10	0%	5	0%	5	0%	63	78	47
HU	HU1	863	86,8	53,6	84,0	40,5	36,2	20	0%	20	45%	10	5%	78	91	65
HU	HU2	1050	94,2	61,0	65,2	35,7	82,1	19	0%	20	5%	10	0%	69	73	64
HU	HU3	622	94,7	61,3	54,2	54,8	74,9	20	0%	20	8%	10	15%	58	68	47
HU	HU4	830	96,2	69,9	87,7	52,4	69,7	20	0%	15	70%	5	0%	74	82	65
HU	HU5	1275	90,4	67,1	65,4	57,4	78,6	20	30%	20	10%	10	0%	83	94	71
IE	IE1	420	95,5	81,0	84,4	67,8	90,8	20	0%	17	0%	9	0%	56	74	38
IE	IE2	220	93,5	71,1	83,3	46,7	83,24	15	47%	20	23%	9	6%	55	60	49
IE	IE3	349	95,5	64,7	83,8	51,4	59	19	21%	13	38%	10	5%	64	75	52
IE	IE4	300	90,2	85,3	94,7	69,7	91,5	10	0%	20	0%	12	0%	58	62	53

Country	Farm	Lact herd size	BTM ab	Cattle ab	NC ab	WC ab	FC ab	NC #	NC BCoV+	WC #	WC BCoV+	FC #	FC BCoV+	BioCheck total	BioCheck external	BioCheck internal
IE	IE5	330	95,2	84,6	89,1	74,1	90,5	17	0%	18	0%	10	0%	59	70	48
IE	IE6	493	93,9	79,7	85,6	62,2	91,4	20	0%	20	30%	10	0%	59	74	43
IE	IE7	220	94,9	67,3	70,3	68,1	63,5	12	0%	20	0%	10	5%	62	75	48
IT	IT1	415	95,1	76,1	88,0	51,2	89,1	20	5%	20	83%	10	0%	45	45	44
IT	IT2	418	90,8	69,3	77,9	46,2	83,7	20	10%	20	25%	10	0%	57	71	42
IT	IT3	171	93,4	66,9	62,7	48,2	89,8	17	0%	20	43%	10	0%	65	81	49
IT	IT4	151	88,3	36,0	44,7	11,7	51,5	18	0%	20	0%	10	0%	46	50	41
IT	IT5	180	91,8	53,7	62,4	42,6	56,2	10	45%	1	100%	10	5%	82	77	66
IT	IT6	298	94,6	64,6	75,7	46,2	71,8	10	60%	10	5%	10	50%	60	64	56
IT	IT7	400	92,3	43,8	56,7	29,8	44,8	11	0%	14	0%	15	0%	83	51	67
IT	IT8	325	94,2	61,4	56,7	58,2	69,4	13	88%	7	100%	10	20%	72	81	62
IT	IT9	190	82,7	54,4	52,8	58,2	52,1	20	85%	20	15%	10	5%	75	90	60
IT	IT10	130	94,1	61,4	49,6	71,1	63,5	4	100%	11	58%	6	0%	81	90	72
NL	NL1	240	86,7	45,9	39,5	41,3	56,9	10	80%	11	100%	7	71%	48	52	44
NL	NL10	245	78	66,2	72,7	36,6	89,2	10	0%	10	0%	5	0%	71	88	53
NL	NL2	250	87,4	58,0	66,5	54,4	53,1	20	0%	20	8%	10	0%	36	33	38
NL	NL3	137	61,3	21,7	26,6	9,1	29,2	12	0%	15	0%	11	0%	58	75	40
NL	NL4	450	90,6	45,2	49,4	21,1	65,1	8	0%	20	0%	7	0%	52	54	49
NL	NL5	265	92,9	60,7	69,0	22,0	91,0	15	0%	15	0%	15	0%	45	49	40
NL	NL6	355	87,6	40,7	68,2	18,8	35,0	10	0%	10	0%	5	0%	65	69	61
NL	NL7	207	76,9	51,2	70,3	23,6	59,7	11	0%	12	0%	9	0%	63	84	42
NL	NL8	250	93,5	18,9	44,6	5,8	6,3	10	5%	10	0%	5	0%	48	58	38
NL	NL9	212	95,1	60,4	71,2	37,2	72,9	10	35%	9	32%	5	0%	70	80	59

Country	Farm	Lact herd size	BTM ab	Cattle ab	NC ab	WC ab	FC ab	NC #	NC BCoV+	WC #	WC BCoV+	FC #	FC BCoV+	BioCheck total	BioCheck external	BioCheck internal
PL	PL10	80	96,2	38,8	33,19	11,63	71,68	10	5%	10	0%	5	0%			
PL	PL2	200	93,9	56,5	42,7	57,9	68,85	10	0%	10	10%	10	0%	70	83	57
PL	PL3	633	85,8	45,1	36,45	52,04	46,75	9	100%	10	40%	10	20%	86	92	80
PL	PL4		96	30,6	51,7	30,53	9,68	10	0%	10	0%	5	0%			
PL	PL5	90	94,1	60,6	60	35,3	86,6	10	80%	10	25%	5	10%			
PL	PL6	380	96,4	56,1	59,6	47,1	61,5	10	0%	10	20%	5	20%			
PL	PL7	650		73,5	72,4	81,7	66,3	10	0%	10	25%	10	0%	76	95	56
PL	PL9	355	88,4	45,7	49,95	34,72	52,49	10	10%	10	20%	10	0%	86	92	80
PT	PT1	338	85,6	43,0	63,8	9,2	56,0	10	0%	10	0%	9	0%	53	62	44
PT	PT2	305	83,9	60,7	59,3	38,4	84,3	9	83%	109	15%	10	25%	63	76	63
PT	PT3	930	94,6	76,3	76,3	63,1	89,6	10	0%	9	47%	10	10%			
PT	PT4	520	91,1	63,7	64,6	43,0	83,6	10	0%	10	21%	6	0%	52	63	41
PT	PT5	180	92,2	49,0	27,0	48,8	71,3	10	75%	10	15%	10	0%	55	76	34
PT	PT7	235	92,2	44,9	46,2	9,8	78,9	10	55%	10	20%	10	0%			
RO	RO1	370	90,7	73,1	81,2	47,4	90,8	20	65%	20	50%	10	0%	65	79	51
RO	RO2	120	89,3	26,6	35,3	7,5	37,2	10	0%	10	0%	10	0%	70	75	65
RO	RO3	235	94,4	75,1	74,3	63,2	87,8	19	72%	20	20%	9	0%	56	65	46
RO	RO4	1000	88,9	82,5	93,2	66,4	87,8	20	0%	20	35%	10	0%	81	90	71
RO	RO5	876	89,0	51,1	73,1	49,6	30,5	20	0%	20	31%	10	0%	61	77	45
SE	SE1	124	96,8	56,0	53,9	63,1	50,9	8	6%	10	15%	5	10%	61	74	47
SE	SE2	340	88,8	59,8	57,3	46,39	75,74	10	0%	10	20%	5	0%	51	64	38
SE	SE3	130	65,1	48,55	44,69	46,26	54,7	11	91%	10	10%	4	20%	64	85	43
SE	SE4	450	91,9	43,48	52,38	9,23	68,82	10	0%	10	5%	5	0%	40	38	41

Country	Farm	Lact herd size	BTM ab	Cattle ab	NC ab	WC ab	FC ab	NC #	NC BCoV+	WC #	WC BCoV+	FC #	FC BCoV+	BioCheck total	BioCheck external	BioCheck internal
SE	SE5	170	91,2	75,8	86,6	47,8	93,0	9	0%	10	20%	5	10%	41	44	37
SK	SK1	2642	85,3	68,7	75,3	50,0	80,7	20	15%	20	23%	10	20%	81	89	72
SK	SK2	550	91,4	33,0	30,9	23,2	45,0	20	5%	20	45%	10	5%	81	88	73
SK	SK3	439	85,2	50,6	61,5	25,9	64,5	20	0%	20	93%	10	10%	67	82	51
SK	SK4	580	78,9	30,2	18,1	43,3	29,1	20	98%	20	18%	10	0%	81	86	76
SK	SK5	700	93,0	72,1	73,1	55,1	88,1	19	45%	20	38%	10	0%	80	90	70
UK	UK1	250	80,5	44,1	46,6	51,0	34,6	13	15%	10	0%	9	0%	43	60	26
UK	UK10	322	92,2	66,9	81,4	46,0	73,3	14	0%	10	65%	10	0%	52	54	49
UK	UK2	820	89,2	49,1	61,6	39,1	46,6	14	21%	15	27%	9	0%	63	76	50
UK	UK3	132	84,0	38,7	29,5	46,1	40,6	4	100%	10	35%	6	8%	45	62	27
UK	UK4	200	87,8	41,1	63,0	18,2	42,0	10	0%	10	0%	10	0%	55	65	44
UK	UK5	450	92,3	37,2	26,3	42,4	42,8	13	31%	9	10%	6	0%	62	70	53
UK	UK6	150	83,7	68,3	71,2	69,0	64,7	9	74%	10	10%	7	0%	32	41	23
UK	UK7	270	78,0	40,0	33,5	47,3	39,3	12	50%	10	20%	9	11%	55	72	38
UK	UK8	210	88,1	76,0	89,9	48,7	89,4	15	3%	10	20%	8	6%	39	38	40
UK	UK9	219	86,7	62,0	50,6	77,0	58,4	14	57%	12	38%	7	14%	51	64	38
